# Dr. Mary Marshall

**Published:** 1910-08-20

**Authors:** 


Dr. Mary Marshall, who died last week at Watford, at the age of seventy-four, was a student of the London School of Medicine for Women, and studied also in Paris and in Edinburgh. She obtained her M.D. degree in Paris in 1879, and in the following year became a licentiate of the Royal College of Physicians, Ireland. Before moving to Watford she practised at Kensington Park and at Upper Berkeley Street. She was senior physician to the New Hospital for Women, Euston Road, and was a member of the Paris Anglo-American Association, the British Medical Association, the Irish Medical Scholars' and Graduates' Association, and the Registered Medical Women's Association, and a contributor to various medical journals.

